# Case Report: Diagnosis and detection of amoxicillin-induced immune hemolytic anemia

**DOI:** 10.3389/fimmu.2026.1773873

**Published:** 2026-03-03

**Authors:** Mengyuan Ding, Yanyan Qin, Jingwei Li, Nina Jiang, Qi Xiao, Xiu Zhou, Guanggang Dou

**Affiliations:** 1 Blood Group Reference Laboratory, Suzhou Blood Center, Suzhou, China; 2 Blood Screening Test Department, Suzhou Blood Center, Suzhou, China; 3 Department of Transfusion, Sichuan Academy of Medical Sciences, Sichuan Provincial People’s Hospital, Sichuan, China; 4 Administration Division, Suzhou Blood Center, Suzhou, China; 5 Clinical Laboratory, Characteristic Medical Center of the Chinese People’s Armed Police Force, Tianjin, China; 6 Clinical Laboratory, Suzhou Hongci Hematology Hospital, Suzhou, China

**Keywords:** amoxicillin, case report, direct antiglobulin test, drug-dependent antibody, drug-induced immune hemolytic anemia

## Abstract

**Background:**

Drug-induced immune hemolytic anemia (DIIHA) is a rare but potentially severe adverse reaction, with antibiotics being common culprits. Amoxicillin, a widely used β-lactam antibiotic, has been infrequently reported to cause DIIHA, posing diagnostic challenges due to its nonspecific presentation.

**Objective:**

This study aimed to report a case of amoxicillin-induced DIIHA, detailing the diagnostic approach and laboratory methods for detecting drug-dependent antibodies.

**Methods:**

A 60-year-old female presented with jaundice, dark urine, and severe anemia following amoxicillin administration. Laboratory investigations included direct and indirect antiglobulin tests, antibody screening and identification, and drug-specific antibody detection using amoxicillin-sensitized red blood cells and drug-added assays. Clinical causality was assessed using the Naranjo Adverse Drug Reaction Probability Scale.

**Results:**

The patient’s direct antiglobulin test was weakly positive for IgG without C3d activation. Serum showed pan-reactivity in saline, which resolved after dithiothreitol treatment, suggesting the presence of IgM cold antibodies. Serum reacted positively with amoxicillin-coated red cells in the indirect antiglobulin test but not with untreated cells, confirming the presence of amoxicillin-dependent antibodies. Hemoglobin and bilirubin levels improved after amoxicillin discontinuation. The Naranjo score was 7, indicating a “probable” association between amoxicillin and hemolysis.

**Conclusion:**

Amoxicillin can induce immune hemolytic anemia through a drug-dependent antibody mechanism. A combination of detailed drug history, serological testing, and drug-specific assays is essential for accurate diagnosis. Immediate withdrawal of the implicated drug remains critical for clinical recovery.

## Introduction

Drug-induced immune hemolytic anemia (DIIHA) is an adverse treatment reaction (1), with an incidence of approximately 2.4/100,000 (2), which can lead to serious complications including a sharp drop in hemoglobin levels, hemoglobinuria, renal failure, and even death (3). Since the first case of DIIHA was reported in 1950, there have been 190 drugs that can induce the body to produce antibodies and cause hemolytic anemia (4), of which nearly half are antibiotics (2, 5). The occurrence of DIIHA relies on antibody production, and drug-induced antibodies can be further categorized into drug-dependent antibodies and drug-independent autoantibodies (6). Drug-dependent antibodies require the presence of the drug to bind to and lyse cells, representing the most common antibodies in DIIHA (7). Drug-dependent antibody reactions have been observed in both penicillin and cephalosporin-induced hemolysis, with the ultimate immune response determined by the bonds formed between the drug and the RBC membrane. Penicillin derivatives form covalent bonds, leading to drug adsorption. The IgG produced by the body targets the drug bound to the RBC membrane, interacting with macrophages and resulting in Fc-mediated extravascular hemolysis (8, 9). In ceftriaxone-induced hemolysis, the loose bonds between the drug and the RBC membrane lead to immune complex formation, activating the complement system and resulting in intravascular hemolysis. The latter mechanism is often associated with worse outcomes and linked to a higher incidence of renal failure, disseminated intravascular coagulation (DIC), and mortality (6). Drug-independent autoantibodies are relatively rare, reacting with and binding to the RBC membrane even in the absence of the stimulating drug, and are serologically indistinguishable from the autoantibodies found in autoimmune hemolytic anemia (AIHA) (9). Amoxicillin belongs to the β-lactam class of antibiotics and is one of the most common penicillin antibiotics in our country, but its inducement of DIIHA is rarely reported (10). In this study, a case of hemolytic anemia caused by amoxicillin antibodies was reported, in order to provide a reference for clinical and laboratory discovery, detection and diagnosis of drug antibodies.

## Materials and methods

### Sample source

The patient, a 60-year-old female, was admitted to the hospital due to “skin itching, bleeding, darkened urine accompanied by jaundice for 3 weeks. She reported receiving intravenous amoxicillin at a local clinic for a cold and fever 3 weeks prior, followed by generalized skin itching and petechiae. A dermatology department at a local hospital diagnosed “drug rash”. Two weeks later, she was sent to the emergency department of a local hospital due to syncope. Her hemoglobin was 48 g/L, platelets were 192×10^9^/L, with darkened urine, jaundice, negative full anti-nuclear antibody panel, normal folate and vitamin B12, elevated ferritin, and haptoglobin <0.1 g/L. The external hospital considered hemolytic anemia. She was treated with hydrocortisone, urine alkalinization, cholagogic and anti-jaundice agents, and blood transfusion. Due to unresolved symptoms, she was transferred to our hospital. The transfusion department detected a positive unexpected antibody screen and a positive direct antiglobulin test (DAT). Blood samples drawn on the day of admission were sent to the Suzhou Central Blood Station Blood Type Reference Laboratory for further antibody identification. The specimens used in this experiment were the remaining samples from this submission. Medical ethics and informed consent complied with basic medical ethical principles.

### Instruments and reagents

Anti-A and Anti-B Blood Grouping Reagents (Monoclonal Antibody) (Lot No. 20231008), Human ABO Blood Group Reverse Typing Red Cell Reagent Kit (Lot No. 20255317), Anti-Human Globulin (Anti-IgG, C3d) Detection Kit (Lot No. 20245001), Anti-Human Globulin (Anti-IgG) Detection Kit (Lot No. 20245101), Anti-Human Globulin (Anti-C3d) Detection Kit (Lot No. 20235202), Panel Cells (Lot No. 20250514), Sample Elution Reagent (Lot No. 20242102), all from Shanghai Blood Biopharmaceutical Co., Ltd. Anti-Human Globulin Test Card (Anti-IgG, Anti-C3d) (Changchun BoXun, Lot No. 20240701), Low-Ionic Strength Anti-Human Globulin Card (BioRad, USA, Lot No. 9209000402), Dithiothreitol (DTT) (Roche, Lot No. 58615529), Papain (BOSF, M0106-25G), L-Cysteine (Ron, 52-90-4), KA-2200 Serology Centrifuge (Kubota, Japan), Microcolumn Gel Incubator (DiaMed, Switzerland), Blood Group Card Centrifuge (DiaMed, Switzerland), TD-A Medical Centrifuge (Changchun BoYan).

### Experimental methods

(1) Blood Group Identification and DAT Test: ABO and RhD blood grouping was performed according to the “National Guide to Clinical Laboratory Procedures (4th Edition)” (11) using the tube method. DAT was performed using both the tube method and the gel card method.

(2) Antibody Identification: Saline antibodies were detected using the tube method. Patient serum treated with 0.01M DTT and acid eluate from patient red blood cells were tested against panel cells in gel cards, following methods 2–21 and 3–16 from the “AABB Technical Manual (20th Edition)” (12), respectively.

(3) Drug Antibody Detection: Methods 4–12 and 4–13 from the “AABB Technical Manual (20th Edition)” were used to detect drug antibodies, employing drug-treated red blood cells and direct addition of the drug to the test system.

- Drug antibody detection using drug-treated red blood cells: Using amoxicillin sodium for injection powder, dissolved in PBS at pH 7.3 to a final concentration of 40 mg/ml. Washed packed red blood cells (DAT-negative) from healthy donors are divided into two tubes. The drug solution is added to one tube, while PBS is added to the other, with a solution-to-packed red cell volume ratio of 15:1. The mixtures are incubated at 37 °C for 1 hour with periodic mixing. After incubation, the cells are washed three times and resuspended in PBS to prepare a 5% suspension. Two sets of tubes are labeled (drug-treated and untreated): Patient serum, acid eluate (obtained according to method 2-21), last wash solution, negative control serum (serum from AB-type donors with a negative antibody screen), and 20-fold dilutions of both patient and negative control sera. Two drops of each specimen are added to the corresponding tubes. To the first set of tubes, one drop of the 5% drug-treated RBC suspension is added; to the second set, one drop of the 5% untreated RBC suspension is added. The tubes are incubated at 37 °C for 60 minutes, centrifuged, and examined for hemolysis and agglutination, with results recorded. Subsequently, the contents of the tubes are transferred to anti-human globulin cards, centrifuged, and examined for agglutination, with results documented. A positive reaction (hemolysis, agglutination, and/or a positive indirect antiglobulin test result) with drug-treated cells, accompanied by a negative reaction with control cells, indicates the presence of drug antibodies.
- Detection of drug antibodies in the presence of the drug: Prepare a 1 mg/ml drug solution in PBS. Label two sets of tubes (untreated and enzyme-treated) for the following mixtures: ① Patient serum + Amoxicillin, ② Patient serum + PBS, ③ Patient serum + Complement (fresh serum) + Amoxicillin, ④ Patientserum + Complement (fresh serum) + PBS, ⑤ Negative control serum + Amoxicillin, ⑥ Negative control serum + PBS. Add 2 drops of the corresponding mixture (e.g., 2 drops serum + 2 drops drug) to each tube. To the first set of tubes, add 1 drop of a 5% suspension of untreated type O red blood cells (fresh RBCs from donor, DAT-negative). To the second set of tubes, add 1 drop of a 5% suspension of enzyme-treated type O red blood cells (enzyme-treated cells prepared according to methods 3–9 to 3-11). Incubate at 37 °C for 1 hour with periodic mixing. Centrifuge and examine for hemolysis and agglutination, recording the results. Then transfer the contents of the tubes to anti-human globulin cards, centrifuge, and examine for agglutination, recording the results. A reactive result in tests containing the patient serum with the drug added, compared to no reactivity in corresponding control tests where PBS replaces the drug, indicates the presence of drug antibodies.

(4) Analysis of Laboratory Indicators: Trends in the patient’s hemoglobin and other laboratory indicators after admission were observed.

(5) Questionnaire on Adverse Drug Reaction Probability: The Naranjo Scale (13) was used to score the probability that the used drug induced the antibodies causing hemolysis.

## Results

**Figure 1:**
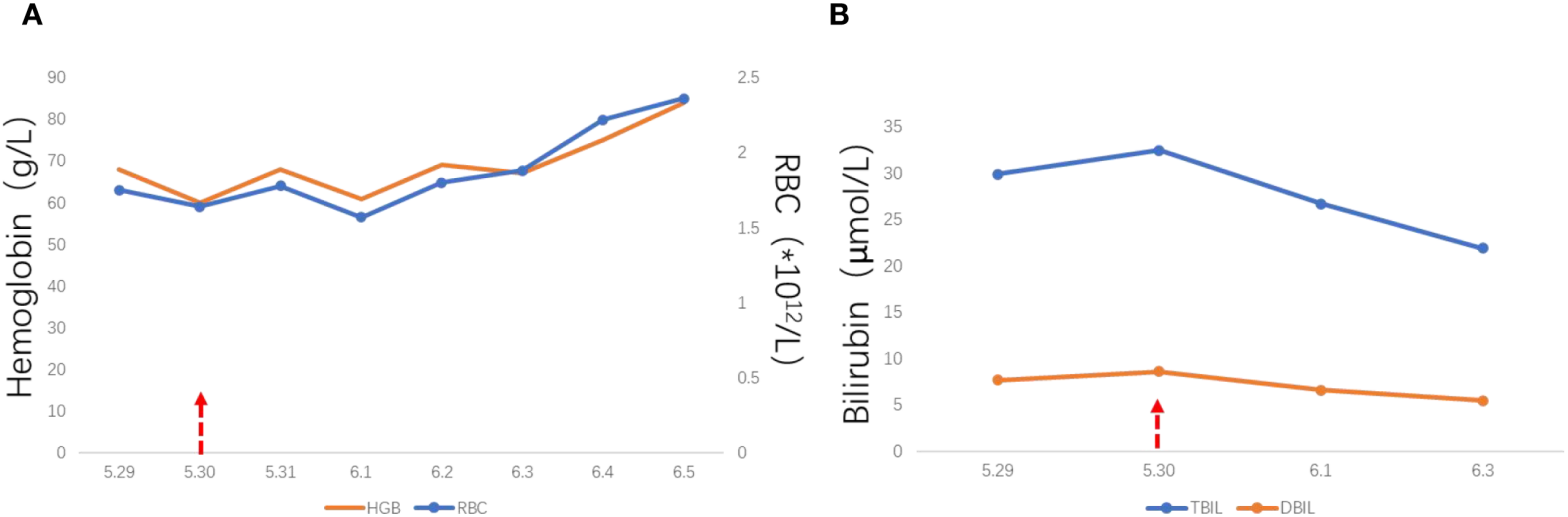
Trends in key laboratory parameters following patient admission. **(A)** Dynamic Changes in Hemoglobin (Hb) and Red Blood Cell Count (RBC): The horizontal axis represents the date after admission (month/day). The left vertical axis indicates hemoglobin concentration (g/L), and the right vertical axis indicates red blood cell count (×10^12^/L). An upward arrow (↑) denotes blood transfusion (May 30). Hemoglobin gradually increased from an initial level of 68 g/L upon admission, while the RBC count showed a corresponding upward trend. **(B)** Dynamic Changes in Total Bilirubin (TBIL) and Direct Bilirubin (DBIL): The horizontal axis represents the date after admission (month/day). The vertical axis indicates bilirubin concentration (μmol/L). Both total bilirubin and direct bilirubin showed a transient elevation in the early phase after admission (May 30) followed by a sustained decline, suggesting gradual resolution of hemolytic activity. All tests were performed using venous blood samples collected at the same time each day.

1. Blood Group and DAT Results: The patient was blood group B, RhD positive. DAT results are shown in **Table 1**. The results indicate that the patient’s red blood cells are sensitized by IgG antibodies, consistent with features of immune-mediated hemolysis, and no significant activation of complement C3d was observed.
2. Antibody Identification Results: The patient’s serum showed pan-reactive patterns with panel cells after immediate spin (IS), with no specificity, and self-control result is positive. After treating the patient’s serum with DTT, the result in the gel card was negative. The acid eluate from the patient’s red blood cells also showed negative reactions with panel cells. Detailed results are shown in **Table 2**. The above results indicate that: ① The antibodies present in the patient’s serum exhibit pan-reactivity in saline medium, which may represent cold antibodies, drug-dependent antibodies, or cold-reactive autoantibodies; ② DTT treatment disrupts IgM antibody activity, and the disappearance of reactivity after treatment suggests that the initial pan-reactivity may be attributed to IgM antibodies; ③ The negative result of the acid eluate indicates that the IgG antibodies sensitized on the red blood cells are not directed against common blood group antigens, further supporting the possibility of drug-dependent sensitization.
3. Drug Antibody Detection Results: To confirm the presence of amoxicillin-related antibodies, detection was performed using both the drug-treated red blood cell method and the drug additive method (as shown in **Tables 3**, **4**).The patient’s serum showed a negative reaction with amoxicillin-treated O cells (Oc) after 37 °C incubation and immediate centrifugation, but showed a weak positive reaction (1+) in the gel card method. No reaction occurred with untreated Oc, indicating the presence of amoxicillin drug antibodies in the patient. These antibodies can recognize and bind to the drug pre-coated on the surface of red blood cells, leading to IgG sensitization, which aligns with the “hapten mechanism” of penicillin-induced immune hemolysis.
4. Analysis of Laboratory Indicators: The patient’s hemoglobin was 68 g/L on the day of admission (May 29). It decreased slightly on May 30 and June 1, but overall, hemoglobin and red blood cell counts showed an upward trend. Total bilirubin and conjugated bilirubin levels increased slightly on May 30 and then showed a downward trend (See **Figure 1**). Reticulocyte percentages were 17.56% on May 31 and 14.78% on June 5. The changes in the above indicators suggest that: ① Mild hemolytic activity persisted during the early stage of admission; ② The hemolytic process gradually ceased after drug discontinuation, with recovery of erythropoiesis and improvement in bilirubin metabolism, which is consistent with the clinical improvement. Notably, the patient’s RBC and hemoglobin levels slightly decreased on May 30th and June 1st. Medication records indicated the initiation of cefepime (6) on May 30th and compound sulfamethoxazole tablets (14) on May 31st. Therefore, the patient’s serum was tested for antibodies against these two drugs, with negative results, indicating no detectable drug antibodies (5). Additionally, since both medications were prescribed as long-term orders and the patient’s hemolytic symptoms showed improvement during their administration, DIIHA caused by these two drugs can be ruled out.
5. Survey results of the probability assessment for adverse drug reaction: The patient scored 7 points (as shown in **Table 5**), which falls into the “probable” category. This scoring outcome further supports, from the perspective of clinical causality, that amoxicillin was the drug responsible for inducing this episode of immune hemolytic anemia.
6. Comparative analysis of amoxicillin-induced immune hemolytic anemia cases: The multiple cases reviewed in this article indicate that amoxicillin-induced DIIHA can manifest with diverse clinical and laboratory features, ranging from the classic hapten/drug adsorption type (such as the high-titer IgG-positive case reported by Gmür et al.) to the immune complex type (such as the C3d-positive case reported by Karunathilaka and Chan Gomez). It is noteworthy that DAT may be negative in some cases, suggesting that non-immune hemolytic mechanisms (such as enzyme deficiency-related hemolysis) should also be included in the differential diagnosis. Regarding laboratory testing, the confirmation of drug-specific antibodies relies on the indirect antiglobulin test using drug-treated red blood cells or the immune complex method. In this series of cases, antibody titers varied widely (from low titers of 2 to high titers of 2000), indicating significant individual differences in immune response intensity. In this case, the DAT was weakly positive for IgG and negative for C3d, consistent with the characteristics of the hapten/drug adsorption type, aligning with most penicillin-induced DIIHA cases. The patient’s hemolysis improved rapidly after drug discontinuation, further supporting the drug as the causative factor. In summary, although amoxicillin-induced DIIHA involves diverse mechanisms, timely recognition, drug withdrawal, and supportive care remain crucial (**Table 6**).

**Table 1:** Patient DAT results.

DAT Method	IgG+C3d	IgG	C3d	Control
Tube Method	0	/	/	/
Gel Card Method	1+	1+	0	0

“+”: Positive, higher number indicates stronger agglutination; “0”: Negative; “/”: Not tested.

**Table 2:** Patient unexpected antibody identification results.

Number	Blood group name	1	2	3	4	5	6	7	8	9	10	Self
Rh-hr	D	+	+	+	+	+	+	0	+	0	+	
	C	0	+	+	+	0	0	0	+	0	+	
	E	+	0	+	+	+	+	0	0	0	+	
	c	+	0	+	+	+	+	+	0	+	+	
	e	0	+	+	+	+	0	+	+	+	+	
Kidd	Jka	+	+	+	0	0	+	0	0	+	+	
	Jkb	0	+	0	+	+	+	+	+	+	0	
MNS	M	0	0	0	+	+	0	+	+	0	+	
	N	+	+	+	0	+	+	+	+	+	+	
	S	0	0	0	+	0	0	0	+	0	0	
	s	+	+	+	+	+	+	+	+	+	+	
	Mur	0	0	0	0	0	0	0	0	0	+	
Duffy	Fya	+	+	+	+	+	+	+	+	+	+	
	Fyb	0	0	+	0	0	0	0	0	0	0	
Diego	Dia	0	0	+	0	0	0	0	0	0	0	
	Dib	/	/	/	/	+	/	/	/	/	/	
Kell	K	0	0	0	0	0	0	0	0	0	0	
	k	+	+	+	+	+	+	+	+	+	+	
Lewis	Lea	0	0	+	+	0	+	0	0	+	0	
	Leb	+	+	+	+	+	+	+	+	0	+	
P	P1	0	0	+	+	+	+	+	0	0	0	
DO	DOa	/	/	/	/	0	/	/	/	0	/	
	Dob	/	/	/	/	+	/	/	/	+	/	
Yt	Yta	/	/	/	/	+	/	/	/	+	/	
	Ytb	/	/	/	/	0	/	/	/	0	/	
Result	IS	2+	2+	2+	1+	1+	2+	2+	2+	2+	1+	2+
	Serum treated with DTT and loaded onto gel cards	0	0	0	0	0	0	0	0	0	0	1+
	Acid-eluted supernatant loaded onto gel cards	0	0	0	0	0	0	0	0	0	0	

“+”: Positive, higher number indicates stronger agglutination; “0”: Negative; IS, Immediate Spin.*.

**Table 3:** Results of drug antibody detection using amoxicillin-treated red blood cells.

Reagent	Amoxicillin-Treated Oc	Untreated Oc
Patient Serum	1+	0
Acid Eluate	0	0
Last Wash Supernatant	0	0
PBS	0	0
Negative-control Serum	0	0
Patient Serum (20-fold dilution)	0	0
Negative-control Serum (20-fold dilution)	0	0

Oc: Red blood cells from group O donor; PBS, Phosphate Buffered Saline.

**Table 4:** Results of drug antibody detection in the presence of amoxicillin.

Reagent	Enzyme-Treated Oc	Untreated Oc
Patient Serum + Amoxicillin	0	0
Patient Serum + PBS	0	0
Patient Serum + Complement (fresh serum) + Amoxicillin	0	0
Patient Serum + Complement (fresh serum) + PBS	0	0
Negative-control Serum + Amoxicillin	0	0
Negative-control Serum + PBS	0	0

**Table 5:** Naranjo adverse drug reaction probability scale score.

Question	Yes	No	Unknown	Patient’s answer	Patient’s Score
1. Are there previous conclusive reports on this reaction?	+1	0	0	Yes	+1
2. Did the adverse event appear after the suspected drug was administered?	+2	-1	0	Yes	+2
3. Did the adverse reaction improve when the drug was discontinued or a specific antagonist was administered?	+1	0	0	Yes	+1
4. Did the adverse reaction reappear when the drug was readministered?	+2	-1	0	unknown	0
5. Are there alternative causes that could solely have caused the reaction?	-1	+2	0	No	+2
6. Did the reaction reappear when a placebo was given?	-1	+1	0	unknown	0
7. Was the drug detected in the blood (or other fluids) in toxic concentrations?	+1	0	0	unknown	0
8. Was the reaction more severe when the dose was increased or less severe when the dose was decreased?	+1	0	0	unknown	0
9. Did the patient have a similar reaction to the same or similar drugs in any previous exposure?	+1	0	0	unknown	0
10. Was the adverse event confirmed by any objective evidence?	+1	0	0	Yes	+1

Total score ≥9 indicates definite causality; total score 5–8 indicates probable causality (supported by objective evidence or quantitative testing); total score 1–4 indicates possible causality; total score ≤0 indicates doubtful causality.

**Table 6:** Comparative analysis of amoxicillin-induced immune hemolytic anemia cases.

Case reference	Age/gender	DAT result (IgG/C3d)	Antibody type	Response model	Antibody titer	Note
Current case	60year/female	IgG weakly positive (1+), C3d negative	Drug Dependence(Drug-Adaptive Type)	Drug-adsorbing type	Not detected	Hemolysis improved after stopping the medication, Naranjo Score: 7
Gmür et al. (1985) (15)	51year/female	IgG strong positive(1:2000), C3d negative	Drug Dependence	Drug-induced (penicillin-type)	Serum 1:128, DAT 1:2000	First reported case of hemolysis after high-dose intravenous use
Rossi et al. (2010) (16)	3year/male	DAT negative (non-immune hemolysis)	Non-immune (enzyme deficiency-related)	Hemolysis caused by oxidative stress	Not applicable	Combined GPI deficiency, DAT negative, non-immune mechanism
Blanquicett et al. (2019) (17)	23year/male	DAT negative, Coombs negative	Non-immune (G6PD deficiency-related)	Hemolysis caused by oxidative stress	Not applicable	G6PD deficiency, no evidence of immune antibodies
Karunathilaka et al. (2020) (7)	53year/male	IgG positive, C3d positive	Drug dependence (immune complex type)	immune complex type	Not detected	
Chan Gomez et al. (2020) (10)	25year/female	IgG negative, C3d positive	Drug dependence (immune complex type)	immune complex type	Not detected	
Sifei Ma et al. (2023) (18)	87year/male	IgG weekly positive, C3d: negative	Drug dependence (drug-adsorptive type)	drug-adsorptive type	Titer: 128	

## Discussion

Amoxicillin is one of the most commonly used β-lactam antibiotics in clinical practice. Reports of amoxicillin-induced drug-induced immune hemolytic anemia are relatively rare, and insufficient clinical awareness often leads to diagnostic delays. Through a typical case, this study systematically demonstrates the diagnostic approach and laboratory verification process for DIIHA, highlighting the value of differential diagnosis, especially in cases with atypical serological presentations.

The diagnostic challenges in this case were primarily reflected in two aspects: first, DAT was only weakly positive for IgG (1+), with an intensity significantly lower than that reported in classic high-titer positive cases, making it prone to being overlooked or misinterpreted as incidental; second, the patient’s serum contained IgM cold antibodies, leading to pan-reactivity in antibody screening and interfering with the identification of specific drug antibodies. These factors complicated the differentiation of this DIIHA case from AIHA and cold antibody syndromes. By employing DTT treatment to eliminate IgM interference and using amoxicillin-sensitized red blood cells for specific detection, the presence of drug-dependent antibodies was ultimately confirmed. This process suggests that for hemolytic cases with weakly positive DAT and nonspecific agglutination, the possibility of drug-related hemolysis should be considered, and targeted laboratory tests should be systematically applied for exclusion and confirmation.

Regarding the pathogenesis, the patient’s serum reacted only with drug-coated red blood cells, with no observed complement C3d activation, consistent with the classic “drug adsorption type” (also known as the “hapten pathway”) mechanism typical of penicillin-class drugs, which primarily leads to extravascular hemolysis. This differs from the “immune complex type” hemolysis (often accompanied by C3d positivity) reported in some cases induced by amoxicillin or combination drugs (such as co-amoxiclav), which can cause more acute intravascular hemolysis and higher associated risks. Additionally, some reports indicate that amoxicillin may trigger non-immune oxidative stress hemolysis in individuals with enzyme deficiencies such as glucose-6-phosphate isomerase (GPI) deficiency, where DAT is negative. The characteristics of this case—weakly positive DAT, IgG type, and absence of complement activation—further enrich the spectrum of serological and clinical phenotypes of amoxicillin-related hemolysis, suggesting potential heterogeneity in its pathogenic mechanisms.

Based on the experience from this case, we recommend the following clinical and laboratory pathway for patients suspected of DIIHA: obtain a detailed history of recent medication use (particularly within two weeks before symptom onset); conduct a comprehensive DAT evaluation, giving due attention even to weakly positive results, and further subtype it (IgG/C3d); if pan-reactivity is observed in serum, employ methods such as DTT treatment to differentiate cold antibody interference; when drug-related hemolysis is highly suspected, perform specific drug antibody testing (e.g., drug-treated red blood cell method), as this is key for differential diagnosis from AIHA; regarding treatment, all suspected drugs should be discontinued immediately, as hemolysis in most patients resolves spontaneously after drug withdrawal without the routine use of corticosteroids.

This study has certain limitations: first, as a single-center case report, caution should be exercised when extrapolating the conclusions. Second, constrained by routine clinical testing, no analysis was conducted on IgG subclasses, affinity, or deeper immunological characteristics of the drug antibodies. Additionally, information such as the timing of medication use prior to admission was retrospective, which may affect the precise assessment of the exposure-response temporal relationship.

In summary, amoxicillin can cause DIIHA characterized by weakly positive DAT, and its diagnosis relies on meticulous medication history investigation, careful interpretation of complex serological results, and specific drug antibody testing. Heightened vigilance for such atypical cases and the establishment of standardized diagnostic protocols are crucial for early recognition, timely intervention, and improved patient outcomes.

## Data Availability

The original contributions presented in the study are included in the article/supplementary material. Further inquiries can be directed to the corresponding authors.
